# Antimicrobial Photodynamic Effect of Cross-Kingdom Microorganisms with Toluidine Blue O and Potassium Iodide

**DOI:** 10.3390/ijms231911373

**Published:** 2022-09-27

**Authors:** Yijun Li, Jingyun Du, Shan Huang, Shaofeng Wang, Yanhuang Wang, Lishan Lei, Chengfei Zhang, Xiaojing Huang

**Affiliations:** 1 Fujian Key Laboratory of Oral Diseases & Fujian Provincial Engineering Research Center of Oral Biomaterial & Stomatological Key Lab of Fujian College and University, School of Stomatology, Fujian Medical University, Fuzhou 350002, China; 2 Restorative Dental Sciences (Endodontics), Faculty of Dentistry, The University of Hong Kong, Hong Kong 999077, China

**Keywords:** antimicrobial photodynamic therapy, potassium iodide, toluidine blue O, *Streptococcus mutans*, *Candida albicans*, early child caries

## Abstract

*Streptococcus mutans* (*S. mutans*) and *Candida albicans* (*C. albicans*) are prominent microbes associated with rapid and aggressive caries. In the present study, we investigated the antimicrobial efficacy, cytotoxicity, and mechanism of toluidine blue O (TBO)-mediated antimicrobial photodynamic therapy (aPDT) and potassium iodide (KI). The dependence of KI concentration, TBO concentration and light dose on the antimicrobial effect of aPDT plus KI was determined. The cytotoxicity of TBO-mediated aPDT plus KI was analyzed by cell counting kit-8 (CCK-8) assay. A singlet oxygen (^1^O_2_) probe test, time-resolved ^1^O_2_ detection, and a ^1^O_2_ quencher experiment were performed to evaluate the role of ^1^O_2_ during aPDT plus KI. The generation of iodine and hydrogen peroxide (H_2_O_2_) were analyzed by an iodine starch test and Amplex red assay. The anti-biofilm effect of TBO-mediated aPDT plus KI was also evaluated by counting forming unit (CFU) assay. KI could potentiate TBO-mediated aPDT against *S. mutans* and *C. albicans* in planktonic and biofilm states, which was safe for human dental pulp cells. ^1^O_2_ measurement showed that KI could quench ^1^O_2_ signals, implicating that ^1^O_2_ may act as a principal mediator to oxidize excess iodide ions to form iodine and H_2_O_2_. KI could highly potentiate TBO-mediated aPDT in eradicating *S. mutans* and *C. albicans* due to the synergistic effect of molecular iodine and H_2_O_2_.

## 1. Introduction

Dental caries represent one of the most prevalent oral diseases and are related to the consequence of metabolic activity of complex microbial biofilm adhered on teeth. Early childhood caries (ECC) is a subtype of dental caries that is highly prevalent in pre-school children and which has gradually constituted a global economical and public health issue [1,2]. It is reported that ECC results in an annual expenditure of more than $120 billion in the United States alone [3]. ECC refers to a unique form of rampant caries that leads to rapid and aggressive destruction of primary teeth. In addition, in some extremely severe cases, children may suffer from chewing problems, pronunciation problems, and aesthetic troubles [4,5].

ECC has multifactorial etiologies, among which cariogenic microbiota-mediated biofilms play a prominent role in the occurrence and development of dental caries. However, current effective approaches to control ECC-associated biofilm are relatively limited, especially in the elimination of notorious cross-kingdom counterparts and their biofilm. Chlorhexidine (CHX) is the most-used antimicrobial agent that can suppress the growth of cariogenic microbes, but is not effective enough against virulent biofilms [6]. Frequent use of CHX has been proven to increase the possibility of microbial resistance and lead to cross-resistance to commonly used antibiotics [7,8]. Many literatures have called for the development of new alternative regimes for the prevention and treatment of ECC [9,10]. Antimicrobial photodynamic therapy (aPDT) is a process that relies on the photoactivation of a non-toxic photosensitizer under an appropriate light source to generate reactive oxygen species (ROS), such as singlet oxygen (^1^O_2_) and free radicals. These highly reactive species could penetrate into microbial cells and interact with the biomacromolecules, leading to rapid inactivation of microorganisms. The benefits of using aPDT include high antimicrobial capacity, and the absence of systemic disturbance and undesirable effects on the surrounding cells or tissues. Therefore, aPDT is used as an auxiliary disinfection method for various oral infections such as periodontitis, peri-implantitis and dental caries [11,12]. The aPDT technique could be applied to decrease the microbial load in saliva and be an adjunct for cavity disinfection procedures. The aPDT presents some advantages to the treatment of ECC when compared with classical antimicrobials, such as high potential for inactivation of various pathogens, lack of resistance even after repeated cycles of treatment, and less required compliance [13,14,15]. A recent clinical study has manifested the actual effectiveness of aPDT in decreasing the colony count of salivary streptococci species in children with severe early childhood caries (SECC) [16].

Nevertheless, there is some debate about whether aPDT-generated ROS could efficiently kill microorganisms, especially in cross-kingdom biofilms. aPDT is still in its primary phase, and there are some obstacles and challenges that are still needed to be overcome. Our previous work also demonstrated that aPDT could inhibit *Streptococcus mutans* (*S. mutans*) growth, but could not achieve a bactericidal effect [17]. In addition, if the concentration of photosensitizer and light dose are continuously increased in order to achieve a stronger antibacterial effect, it may have an adverse effect on the surrounding tissues. Thus, extensive efforts have been attempted to improve aPDT efficiency, one of which is introducing non-toxic inorganic salts into the aPDT procedure [18]. Potassium iodide (KI) is the most studied, which is accepted as an antifungal agent in clinical medicine. Accumulating experimental studies have demonstrated that the combination of simple nontoxic KI solution with methylene blue (MB) or rose bengal (RB), subsequently irradiated by light, could give rise to enhanced microbial killing when compared to using photosensitizers alone [19,20]. The potentiated antimicrobial effect of KI could avoid the staining effect caused by the use of high-concentration photosensitizer in dentistry applications [21].

To date, a few studies have already demonstrated the potential effect of the combination of KI and aPDT against oral pathogens [22,23]. To our knowledge, there is a fundamental difference in susceptibility to aPDT among microorganisms even in a similar oral microenvironment [24,25]. Toluidine blue O (TBO) is a cationic photosensitizing agent with many benefits, including low excitation energy, relatively low cost, and high transmembrane permeability in comparison with other phototherapeutic agents. Furthermore, it is one of the FDA-approved photosensitizers in clinical medicine. Currently, no data is available on the combination of KI with TBO-mediated aPDT in cross-kingdom microorganisms. In order to gain a comprehensive understanding of the specific effects produced by the combination of KI with TBO-mediated aPDT, the purpose of this study was to evaluate the photodynamic effect of TBO in the presence of KI in the inactivation of cross-kingdom microorganisms and investigate its involved mechanisms.

## 2. Results

### 2.1. Assessment of Antimicrobial Effect of KI

**Figure 1:**
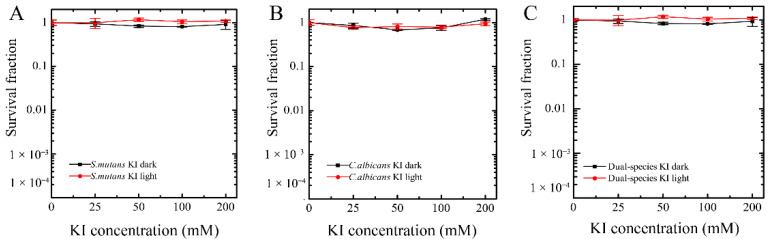
Assessment of dark toxicity and phototoxicity of potassium iodide (KI) on plaque suspension. The survival fraction of *S. mutans* (**A**), *C. albicans* (**B**), and total microorganisms (**C**) were determined by the counting forming unit (CFU) method.

In order to confirm that the potentiation effect of KI was not due to its antimicrobial ability per se, we first evaluated the dark toxicity and phototoxicity of KI on plaque suspension. According to Figure 1A, a series of concentrations of KI neither at dark nor with light illumination displayed killing on total microorganisms in plaque suspension. We also used selective agar plate for counting to assess the actual effect of KI on these two microorganisms, and found that KI had no killing effect on *S. mutans* and *Candida albicans* (*C. albicans*) at dark or light illumination (Figure 1B,C).

### 2.2. aPDT Parameters Exploration

#### 2.2.1. KI Concentration

**Figure 2:**
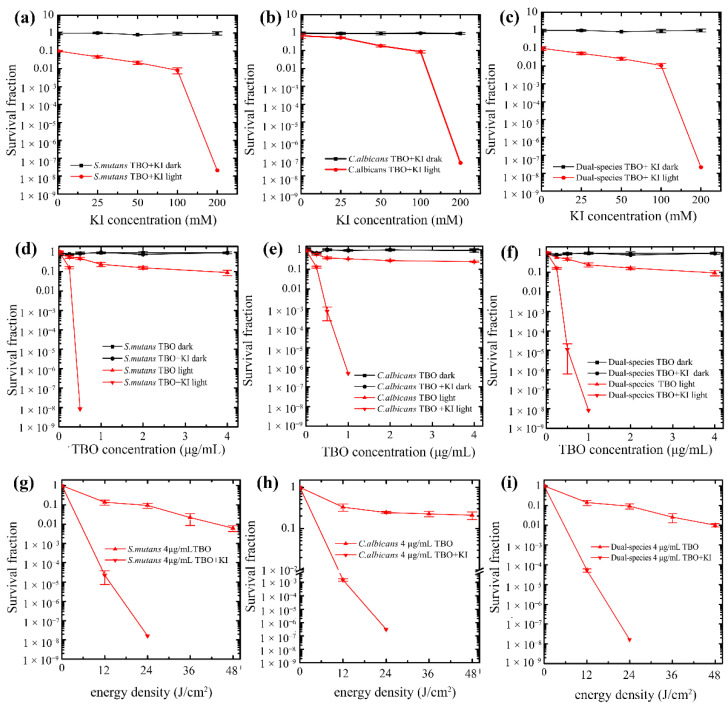
Antimicrobial effect of different concentrations of KI on toluidine blue O (TBO)-mediated antimicrobial photodynamic therapy (aPDT) on microorganisms in plaque suspension. The survival curves of (**a**) *S. mutans*, (**b**) *C. albicans,* and (**c**) total microorganisms. Antimicrobial effect of different concentrations of TBO with or without KI on microorganisms in plaque suspension. The survival curves of (**d**) *S. mutans*, (**e**) *C. albicans*, and (**f**) total microorganisms. Antimicrobial effect of different light doses on microorganisms in plaque suspension. The survival curves of (**g**) *S. mutans*, (**h**) *C. albicans*, and (**i**) total microorganisms.

We used 4 μg/mL TBO in this experiment, because if the TBO concentration is too high, it would cause significant killing and obscure the potentiated effect of KI. According to our pre-experiment results, 4 μg/mL TBO-mediated aPDT could result in a 1 log_10_ reduction of microorganism numbers in plaque suspension. Next, we used a series of concentrations of KI up to 200 mM to investigate the dependence of antimicrobial efficiency on KI concentration. Figure 2a–c show the survival curves of the total microorganisms, *S. mutans,* and *C. ablicans* in plaque suspension, respectively, when treated with TBO and KI. There was no dark toxicity for microorganisms when in incubation with TBO and KI. There was still a slight decrease in living microorganism numbers even when the concentration of KI reached 100 mM. Interestingly, we found that the addition of 200 mM KI could highly potentiate TBO-mediated aPDT from eradicating the total microorganisms completely in plaque suspension. It can be observed that the effect of KI potentiation was more obvious in *S. mutans* rather than *C. ablicans* (Figure 2b,c).

#### 2.2.2. TBO Concentration

To verify that the microbial inactivation was also dependent on the TBO concentration, we treated microbial cells with a range of 0.25 to 4 μg/mL TBO plus 200 mM KI. The results in Figure 2d–f represent the survival fraction of the total microorganisms, *S. mutans*, and *C. ablicans* in plaque suspension. As TBO concentration increased, there was a very slight increase in microbial killing when cells were incubated only with TBO. However, in the presence of KI, increasing TBO concentration from 0.25 to 4 μg/mL presented a remarkable killing effect against two strains in plaque suspension. In addition, this increased killing effect was more pronounced than the groups that were merely incubated with TBO. The results also showed the eradication of *S. mutans* in plaque suspension was achieved in 0.5 μg/mL TBO-mediated aPDT plus KI, while the full elimination of *C. ablicans* was observed with 1 μg/mL TBO-mediated aPDT plus KI (Figure 2e,f).

#### 2.2.3. Light Dose

The energy density dose is also a determinant factor in the antimicrobial effectiveness of aPDT. We had used a fixed dose of 24 J/cm^2^ in our previous experiment according to the manufacturer’s instructions, but we wanted to know how much the final microbial killing depended on the amount of light dose. Hence, we tested a wider range of light sources. We employed a wide range of doses (12, 24, 36, 48 J/cm^2^) that corresponded to illumination times (30, 60, 90, 120 s). All groups were treated in one time illumination regardless of energy densities. In Figure 2g–i, the results show that increasing the light dose, as expected, induced more killing not only in TBO-mediated aPDT, but also in TBO-mediated aPDT plus KI.

### 2.3. Cytotoxicity Evaluation

**Figure 3:**
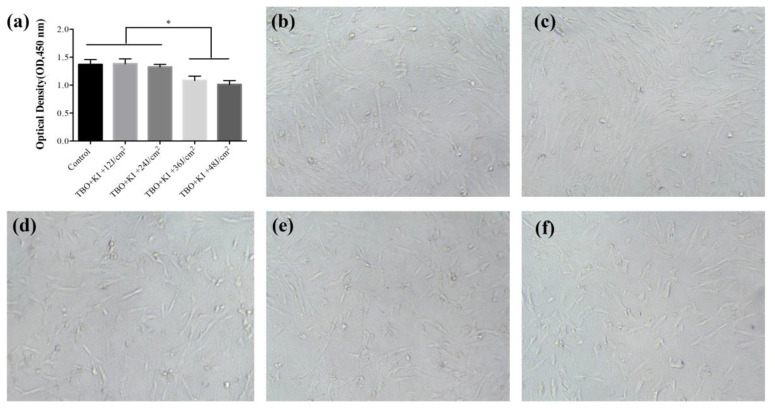
Effect of TBO-mediated aPDT with KI on human dental pulp cells (hDPCs) viability and morphology. HDPCs were incubated with 1 μg/mL TBO and 200 mM KI after shining different doses of red light. (**a**) HDPCs viability in each group determined by cell counting kit-8 (CCK-8) assay. (**b**) Light microscope observation of hDPCs without any treatment, magnification ×100. (**c**–**f**) Light microscope observation of hDPCs when incubated with 1 μg/mL TBO and 200 mM KI after shining 12, 24, 36, 48 J/cm^2^ of red light respectively, magnification × 100. (* *p* < 0.05 in ANOVA test).

Figure 3 displays the cell viability and morphology after treatments. As shown in Figure 3a, when human dental pulp cells (hDPCs) were incubated with 1 μg/mL TBO plus KI and received 12 J/cm^2^ or 24 J/cm^2^, there was no significant difference of cell viability compared to the control group (*p* > 0.05). As light dose increased, slight cytotoxicity could be observed and the survival rate was approximately 80% in the higher-doses group. The morphology of cells in the experimental groups were similar to the control groups, without any irregular shape (Figure 3b–f).

### 2.4. ^1^O_2_ Detection and ^1^O_2_ Quenching Experiment

**Figure 4:**
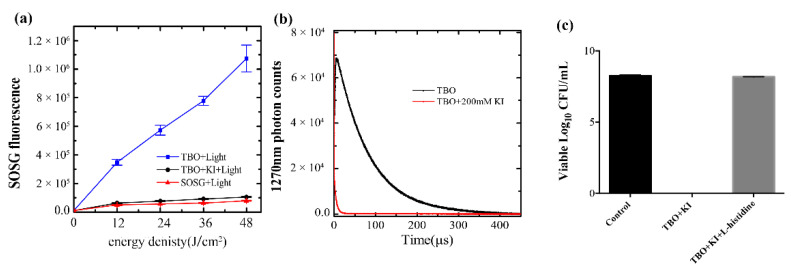
(**a**) ^1^O_2_ detection by singlet oxygen sensor green (SOSG) fluorescent probe. (**b**) The time-resolved kinetics of the formation and decay of ^1^O_2_ 1270 nm phosphorescence. (**c**) Assessment of the antimicrobial effect of TBO-mediated aPDT in combination with KI in the presence of 50 mM L-histidine.

TBO-mediated aPDT could result in singlet oxygen sensor green (SOSG) activation, which indicated that TBO may be more likely to operate via Type II mechanism to generate ^1^O_2_ (Figure 4a), while the quenching of SOSG fluorescence was observed during the photoactivation of TBO plus KI. Representative time-resolved ^1^O_2_ luminescence spectrum detected in TBO with or without 200 mM KI is shown in Figure 4b. The observable ^1^O_2_ lifetime in TBO photoactivation was longer than the sample treated with TBO photoactivation plus KI. Figure 4c shows that 1 μg/mL TBO-mediated aPDT could eradicate the microorganisms from plaque suspension in the presence of 200 mM KI. However, when 50 mM L-histidine was added, there was no significant difference in microorganism numbers when compared to the control group (Figure 4c).

### 2.5. Free Iodine Molecules and H_2_O_2_ Detection

**Figure 5:**
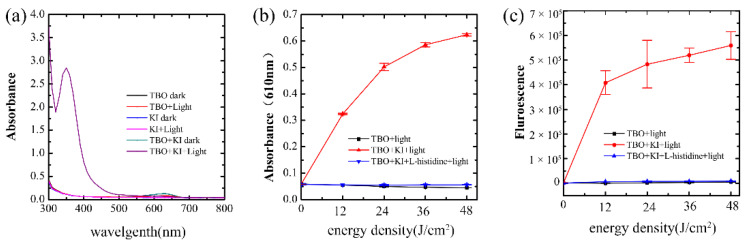
(**a**) The spectrum of different reaction mixtures at dark and after illumination. (**b**) Production of iodine measured by starch indicator assay. (**c**) Production of hydrogen peroxide (H_2_O_2_) measured by Amplex Red assay.

According to the Uv-vis spectrum, there is no difference when KI was under dark or light illumination (Figure 5a). Photobleaching of TBO after light illumination was detected (Figure 5a). However, there was a new sharp peak at 350 nm in the solution of TBO with KI after light delivery (Figure 5a). The iodine starch assay result also confirmed the generation of free iodine molecules in the solution of TBO plus KI after illumination (Figure 5b). It can be observed that a higher amount of iodine molecules was produced in the solution with increasing light dose, and the addition of 50 mM L-histidine completely abolished the production of free iodine (Figure 5b).

The results of the Amplex Red assay are illustrated in Figure 5c. When 1 μg/mL TBO was excited by red light, there was very little generation of H_2_O_2_. The addition of KI to TBO produced more H_2_O_2_ compared to TBO-mediated aPDT. Similarly, the addition of 50 mM L-histidine completely quenched the production of H_2_O_2_.

### 2.6. Anti-Biofilm Assessment

**Figure 6:**
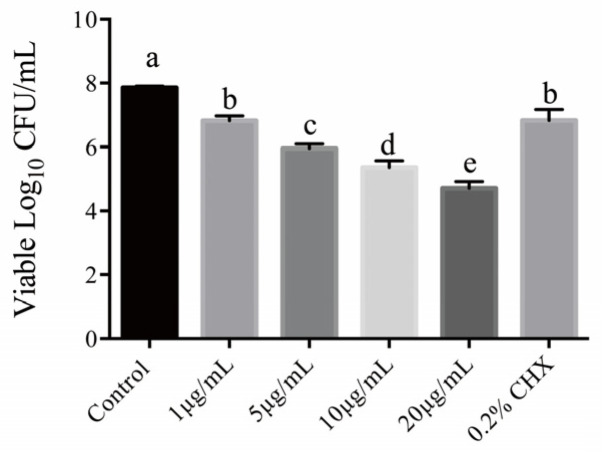
Colony-forming units of living microorganism on dentin slices after different treatments. Values with dissimilar letters indicated significant difference between two groups (*p* < 0.05).

The disinfection of dual-species biofilm on dentin slices is illustrated in Figure 6. All experimental groups and the 0.2% CHX group presented higher disinfection ability than the PBS group, with a lower level of living microorganisms on the dentin slice (*p* < 0.05). Similar to the above previous results, a TBO concentration-dependent trend was also observed in aPDT plus KI against biofilm. The reduction in total counts of two strains with 1 μg/mL TBO, 5 μg/mL TBO, 10 μg/mL TBO and 20 μg/mL TBO were 1.03 log_10_, 1.89 log_10_, 2.48 log_10_ and 3.13 log_10_, respectively. Apart from 1 μg/mL TBO group, the remaining living counts of other combined groups using TBO and KI were significantly lower than those of in the 0.2% CHX group.

## 3. Discussion

Our study showed that KI could dramatically improve the disinfection effects of TBO-mediated aPDT on cross-kingdom microbes. So far, this is the first in vitro study evaluating the antimicrobial effects of TBO-mediated aPDT against cross-kingdom microbes in the presence of KI, also shedding light on the mechanisms responsible for the enhanced antimicrobial activity.

It is acknowledged that ECC is a polymicrobial infectious disease resulting from dysbiosis of the microbial ecology in the oral cavity. *S. mutans* is regarded as the main pathogenic bacteria and contributor in ECC lesions, as it can decompose dietary sucrose into organic acids and produce abundant extracellular glucans to form biofilms and provide sites for other microorganisms’ adherence [4,26]. Recently, from clinical evidence, high amounts of *C. albicans* were frequently detected together with *S. mutans* in the plaque-biofilm formed on the tooth surface in ECC children [27,28]. The cross-kingdom interaction between *S. mutans* and *C. albicans* could enhance the matrix formation and modulate the virulence expression of biofilms, consequently leading to tooth decay and rampant caries [29,30]. The disruption and eradication of cross-kingdom microbes remains an important challenge in ECC treatment. Therefore, in the present study, the mixture of *S. mutans* and *C. albicans* was used.

aPDT is a process that involves the photochemical reaction among a photosensitizer, light and oxygen, and many variables should be considered in order to assure its effectiveness. In this study, KI concentration was also investigated as a variable to predict treatment effectiveness. Our results showed that the addition of 200 mM KI to TBO, and subsequent excitement with red light could trigger microorganism reduction dramatically. Vecchio et al. reported that 100 mM KI could significantly potentiate the antibacterial effect of methylene blue MB-mediated aPDT against *Staphylococcus aureus* and *Escherichia coli* [20]. In the study conducted by Huang et al., it was pointed out that BB4-PPBA photoactivation can be highly potentiated against *C. albicans* with the addition of 400 mM KI [31]. There may be differences between the KI concentration used in the above research results and this study due to the photosensitizer used. Different types of photosensitizers harbor different features, such as ^1^O_2_ production, binding ability with microorganisms, charge number and distribution, which may influence the reaction between the photosensitizer and KI.

To ensure maximum microorganism reduction, higher concentrations of photosensitizer or light doses were explored in previous studies, since they are important parameters for determining aPDT outcomes [32,33]. However, it cannot be ignored that higher TBO concentration after photoactivation was reported to induce cytotoxicity to normal host cells [34]. Likewise, high concentration of photosensitizer after incubation may stain the tooth dentin and affect the aesthetics of anterior teeth in dental treatment [35]. Thus, a lower concentration of TBO was used in this study. Our data revealed that by fixing two of the three parameters, TBO concentration and light dosage are directly proportional to the final antimicrobial effects, not only in TBO-mediated aPDT but also TBO-mediated aPDT plus KI, which was in line with prior investigations. Surprisingly, 1 μg/mL TBO at plus KI after shining 24 J/cm^2^ red light could eradicate *S. mutans* and *C. albicans* from plaque suspension.

Before applying a new antimicrobial strategy into clinical medicine, in vitro studies should be carried out to test not only its high antimicrobial efficiency but also its biocompatibility. It is verified that high concentrations of photosensitizers or light intensity application can lead to some apoptotic and necrosis effects in cells [36]. hDPCs are sensitive to external stimulus, and it has been demonstrated that if the temperature rises over 5.5 °C after illumination, this can induce reversible damage to the pulp [37]. The evaluation of cell cytotoxicity in this study showed the excellent potential of TBO-mediated aPDT plus KI in sterilizing microorganisms without affecting hDPCs viability. Though there was a slight effect on hDPCs viability at high light doses, it could be diminished by the underlying dentin and pulp tissue physiology.

The potentiation effect on microbial killing during TBO photoactivation due to the presence of KI prompted us to elucidate the involved synergy mechanism. Scholars proposed that ROS generated by the aPDT procedure could interact with KI to generate iodine species (iodine radicals and free iodine) in two ways [18,38]. One is that the triplet state photosensitizer would undergo electron transfer to excess iodine ions in solution to form the photosensitizer radical cation and the iodine radical anion. The other is the interaction between ^1^O_2_ and KI to form H_2_O_2_ and molecular iodine. TBO has been demonstrated to operate via a Type II mechanism by generating ^1^O_2_, which was highly reactive. For this reason, we speculated that ^1^O_2_ produced by TBO-mediated aPDT may act as a principal mediator to oxidize iodine ions to free iodine and H_2_O_2_. To confirm our assumptions, we investigated whether the addition of KI would quench the ^1^O_2_ production. The SOSG probe result and ^1^O_2_ time-resolved detection confirmed our assumption; the activation of SOSG and ^1^O_2_ 1270 nm luminescence signals were both quenched in the presence of KI. Additionally, L-histidine as a ^1^O_2_ quencher was added into the reaction mixture and completely inhibited microbial killing. The reason for selecting L-histidine as a quencher instead of azide was that azide would enhance TBO-mediated aPDT killing [39], which may confuse the results. The above results revealed that the potentiated antimicrobial effect is due to the interaction of ^1^O_2_ and KI. The previous literature reported the association of an abrupt decrease in the survival profile and the high production of iodine [40], which was also observed in our study. According to our mechanistic experiments, TBO-mediated aPDT plus KI generate free iodine and H_2_O_2_. As reported earlier, a one-electron transfer reaction from iodine to ^1^O_2_ to give superoxide and iodine radical is unlikely to occur in thermodynamics [41]. The proposed mechanism is that an initial addition reaction generates peroxyiodide via the oxidation of iodide by ^1^O_2_, then peroxyiodide decomposes into iodine and H_2_O_2_ [42]. Similar mechanisms were also observed in several photosensitizers that act mainly through the Type II process when in combination with KI [19,43]. In the present study, we also detected the generation of H_2_O_2_. H_2_O_2_ is commonly used as a sterilization agent, disinfectant, and antiseptic in dental practice. The combination of iodine and H_2_O_2_ has been demonstrated to be more effective in antimicrobial ability in comparison with either compound alone. Owing to this mechanism, coupling TBO-mediated aPDT with KI led to the improved antimicrobial effect on dual-species biofilm, which would allow to reduce the concentration of TBO used and therefore its side effects. Following the notion of minimal invasiveness, modern dentists prefer to conduct select grinding techniques when excavating deep caries. However, the residual microorganisms hidden in the inner layer, especially the robust biofilm, often lead to treatment failure. The existence of the *C. albicans* and *S. mutans* cross-kingdom biofilm is associated with caries recurrence in ECC [27]. According to the findings observed in this study, aPDT in combination with KI could be used for cavity disinfection during cavity preparation by deactivating the remaining microorganisms in the demineralized inner layer, therefore preventing the occurrence of secondary caries and improving the therapeutic outcome. In addition to the cavity disinfection, the protocol could be used to prevent white spot lesions and tooth decay in orthodontics patients by reducing the microorganism load on brackets. The protocol is more promising than aPDT alone in caries prevention because it could not only achieve an antibacterial effect but also could avoid the high concentration of the photosensitizer used.

## 4. Materials and Methods

### 4.1. Microorganism Growth and Culture Condition

The experiments were performed using *S. mutans* UA159 and *C. albicans* ATCC10231 strain. *S. mutans* was maintained on brain heart infusion (BHI, OXIOD, Basingstoke, UK) agar plates anaerobically at 37 °C, whilst *C. albicans* was cultivated aerobically on sabouraud dextrose agar plates (Hopebio, Qingdao, China) at 37 °C. After 48 h, the microorganisms were transferred to the broth media and incubated at 37 °C until the mid-logarithmic phase.

### 4.2. Dual-Species Plaque Suspension Preparation

Before inoculum, the microorganisms were collected by centrifugation (4000× *g*, 5 min, 4 °C) and resuspended in phosphate-buffered saline (PBS). Suspensions of microorganisms were standardized to OD values equal to 2.5 × 10^7^ CFU/mL for *S. mutans* and 2.5 × 10^6^ CFU/mL for *C. albicans* using a microplate reader (SpectraMax iD3, Molecular Devices, CA, USA). Equal volumes (10 μL) of each microorganism and 180 μL tryptone-yeast extract (TYE; 2.5% tryptone and 1.5% yeast extract) broth supplemented with 1% sucrose were incubated into 96-well microtiter plates to form dual-species biofilms. The dual-species biofilm was cultured for 48 h at 37 °C and the medium was changed at 24 h. After growth, 200 μL PBS was added to each wall to disperse the biofilm and obtain dual-species plaque suspension for the following experiment.

### 4.3. Photosensitizer and Light Source

TBO (Sigma-Aldrich, St. Louis, MO, USA) was used as a photosensitizer and was prepared in PBS. KI (Sigma-Aldrich, St. Louis, MO, USA) was dissolved in distilled water. Both stock solutions were prepared immediately and filtered by 0.22 μM syringe filters before usage. The light source was a red LED light source (Denfotex, Redhill, UK) which centered at 635 nm with a bandwidth of 10 nm. The radiation distance between the light to the bottom of the plates was set at 1 cm. The output power was 530 mW and the beam diameter was 1.3 cm. Power measurements were measured with a power meter (Ophir Optronics Ltd., Danvers, MA, USA). The light dosages used in this study (12, 24, 36, 48 J/cm^2^) corresponded to illumination times (30, 60, 90, 120 s).

### 4.4. Assessment of Antimicrobial Effect of KI

Resuspended dual-species plaque suspension (200 μL) in PBS was incubated with different concentrations of KI (200 μL) in a 48-well plate in the dark at room temperature. An aliquot of 200 μL from each well was incubated at dark for 30 min which was used as the dark control, and another aliquot (200 μL) was incubated and irradiated with a 24 J/cm^2^ dose of red light. After treatments, the aliquots from each well were serially diluted immediately and plated on agar plates. For species isolation, mitis salivarius agar (MSA, TOPBIO, Yangtai, China) supplemented with 0.2 U/mL bacitracin (TOPBIO, Yangtai, China) was used for *S. mutans* counting, and Chromogenic Candida Agar (OXIOD, Basingstoke, UK) was used for *C. albicans* counting.

### 4.5. aPDT Parameters Exploration in Dual-Species Plaque Suspension

The dual-species plaque suspension obtained was used to study the relationship between different parameters and antimicrobial effect. The following parameters were investigated to find a dose-dependent relationship: (1) KI concentration at four levels: 25, 50, 100, and 200 mM; (2) TBO concentration ranging from 0.25 to 4 μg/mL at five levels, 0.25, 0.5, 1, 2, and 4 μg/mL; and (3) energy density at four levels: 12, 24, 36, and 48 J/cm^2^. We generally fixed two of the three parameters and changed the last parameter to optimize the approximate parameter. The details of each experiment are shown in Table 1, Table 2 and Table 3. The plaque suspension (200 μL) was incubated with TBO (100 μL) and KI (100 μL) in a 48-well plate at dark for 30 min. Next, the wells were exposed to red light according to the manufacturer’s instructions. After illumination, the aliquots were serially diluted and plated as before.

### 4.6. Cytotoxicity Assay

The three molar teeth were extracted and immediately kept in cold sterile Hanks’ balanced salt solution (Invitrogen, Carlsbad, CA, USA). The tooth was fractured into several parts by bone forceps under sterile conditions, and the dental pulp tissue was isolated and collected. The pulp tissue was minced into small pieces and digested with a mix of 3 mg/mL type I collagenase and 4 mg/mL dispase. The isolated cells were cultured in α-minimum essential medium (α-MEM, Hyclone, Logan, Utah, USA) supplemented with 10% fetal bovine serum (FBS, Gibco, CA, USA) and 1% penicillin/streptomycin (Solarbio, Beijing, China) at 37 °C in humidified atmosphere of 5% CO_2_.

hPDCs from the third or fourth passage were used for the cytotoxicity experiment. hDPCs were seeded in 96-well plates with α-MEM containing 10% FBS at a density of 1 × 10^4^ cells per well. When cell confluence reached nearly 70–80%, cells in experiment groups were treated with TBO (1 μg/mL) plus KI (200 mM) for 30 min in dark and received different dosages of red light (12, 24, 36, 48 J/cm^2^). The cells without any treatment were set as control. After treatments, the cells were washed with PBS and replaced with a fresh culture medium for another culture of 24 h. Next, cell viability in each group was measured by cell counting kit-8 (CCK8, Dojindo, kumamoto, Japan). Each well was incubated with 10 μL CCK-8 with 190 μL α-MEM at 37 °C for 120 min. After incubation, the optical density was determined at 450 nm on a microplate reader. The cell morphology in each group was observed by an inverted light microscope (Olympus, Tokyo, Japan).

### 4.7. SOSG Probe Test

Cell-free fluorescent-probe experiments were performed using a 96-well dark plate. 1 μg/mL TBO with or without 200 mM KI, and SOSG probe (Invitrogen, Carlsbad, CA, USA) at a final concentration of 1 μM were added to each well. The wells were exposed to different time intervals to receive sequential doses, starting at 12 and going up to 48 J/cm^2^. The fluorescence intensity of each well was determined immediately by a microplate reader after an incremental fluence was delivered. The fluorescence excitation wavelength was 505 nm and the emission wavelength was 525 nm.

### 4.8. Time-Resolved ^1^O_2_ Detection

The detection of ^1^O_2_ luminescence was kindly assisted by Pro. Lin, Fujian Normal University, China. The developed detection system was described previously in detail elsewhere [44]. The solid-state AOM Q-switched laser at 671 nm (AO-V-671, Changchun New Industries optoelectronics Tech, Changchun, China) was used as the excitation light source with a frequency of 10 kHz. D_2_O solutions of TBO with or without KI were placed in a 10 mm optical path quartz fluorescence cuvette. The laser light passed through a splitter and was directed into the measured solution. The exciting luminescence was collected at 90° to the excitation beam through a 1000 nm long-pass filter (Omega Optical, Brattleboro, VT, USA), and the collection optics and the narrow-band filter centered at 1270 nm that were placed in front of the photomultiplier tube (PMT) (H10330-45, Hamamatsu Corp, Hamamatsu, Japan). The PMT output pulses were amplified and passed to a pulse-height discriminator in the photon counter.

### 4.9. ^1^O_2_ Quencher Experiment

To further investigate whether the potentiated effects of KI addition were attributable to the ^1^O_2_ generated by TBO-mediated aPDT, L-histidine as a ^1^O_2_ quencher was added to the reaction mixture including KI, TBO, and plaque suspension. Briefly, the dual-species plaque suspension was incubated with TBO (1 μg/mL) plus KI (200 mM) and/or the addition of L-histidine (50 mM) at dark for 30 min and exposed to 24 J/cm^2^ red light. Plaque suspension without any treatment was set as the control group. After the irradiation, the number of CFU/mL in the plaque suspension was determined as described above.

### 4.10. UV-Vis Spectrum Analysis

A UV–vis spectrophotometer was used to obtain the absorption spectrum of 1 μg/mL TBO and 200 mM KI, and spectra were measured before and after delivering 24 J/cm^2^ of light. The groups were divided into six groups, including (1) TBO dark, (2) KI dark, (3) TBO + KI dark, (4) TBO + light, (5) KI + light and (6) TBO + KI + light. For dark groups, the solution was kept at dark for 30 min, while for the light groups, the solution was kept at dark and then received 24 J/cm^2^ illumination.

### 4.11. Iodine Starch Test

In order to explore whether iodine molecules are produced in the combination of KI with TBO-mediated aPDT, and whether L-histidine can prevent the production of iodine molecules, an iodine starch test was performed. The groups were (1) TBO + light, (2) TBO + KI + light and (3) TBO + KI + L-histidine + light. The concentrations of TBO, KI, and L-histidine were the same as previously. The reaction mixture was kept at dark and then received sequential doses of illumination. Aliquots (50 μL) of each sample were withdrawn after light delivery and added to the starch indicator (50 μL). A microplate reader was used to measure incremental absorbance at OD_610_ nm.

### 4.12. Amplex Red Assay for H_2_O_2_

In order to verify whether H_2_O_2_ was generated in the combination of KI with TBO-mediated aPDT, and whether L-histidine can prevent the production of H_2_O_2_, Amplex Red hydrogen peroxide/peroxidase assay was performed. The groups included (1) TBO + light, (2) TBO + KI + light, and (3) TBO + KI + L-histidine + light. The treatment was as previously described. After light delivery, 50 μL of each sample was withdrawn and added into 50 μM Amplex Red reagent (Thermo fisher, Carlsbad, CA, USA) and 0.1 U/mL horseradish peroxidase (HRP). The reaction mixture was incubated at dark for 30 min and measured by a microplate spectrophotometer. The fluorescence excitation wavelength was 505 nm and the emission wavelength was 525 nm.

### 4.13. Anti-Biofilm Assessment by CFU Assay

The dual-species biofilm was cultured on bovine dentin slices (7 × 4 × 1.2 mm) which were placed in a vertical position in 24-well culture plates. The inoculation ratio and biofilm growth cycle were the same as previously. After biofilm formation, the medium was discarded, and wells were rinsed with 200 μL PBS to remove planktonic microorganisms. In this part, we employed a higher concentration of TBO in testing the anti-biofilm potential of TBO-mediated aPDT in combination with KI. The dentin slice was incubated with equal volumes of different concentrations of TBO (1, 5, 10, 20 μg/mL) and KI (100 mM) at dark for 30 min. Subsequently, the dentin slice was irradiated by an LED light source for 60 s, corresponding to a fluence of 24 J/cm^2^. Additionally, the dentin slice was immersed in PBS or 0.2% CHX for 60 s set as negative group and positive group, respectively. After treatments, each sample was resuspended in 1 mL PBS, serially diluted and plated on agar plate for CFU counting.

### 4.14. Statistical Analysis

CFU counts were log-transformed for statistical analysis. One-way analysis of variance (ANOVA) followed by Tukey HSD were performed by SPSS (IBM SPSS 21.0, IBM, Armonk, NY, USA) to analyze CFU data and cytotoxicity data. The significance level α was set at 0.05.

## 5. Conclusions

Taken together, our results showed KI could highly potentiate TBO-mediated aPDT not only in planktonic state but also in biofilm due to the synergistic effect of newly formed molecular iodine and H_2_O_2_, suggesting it could be an alternative method to treat oral infections caused by cross-kingdom microorganisms such as ECC. The combination of TBO-mediated aPDT with KI is predicted to be useful for cavity disinfection or prevention of tooth decay during caries treatment. However, our study is preliminary research toward investigating the effectiveness of TBO-mediated aPDT plus KI as a potential alternative treatment for cross-kingdom microbes. To allow broader clinical applications, its capacity in the disinfection of different strains or even resistant strains, followed by in vivo infection models are needed to be carried out in our further study before translating it into practical situations.

## Figures and Tables

**Table 1:** Parameters for varying potassium iodide (KI) concentration on toluidine blue O (TBO)-mediated antimicrobial photodynamic therapy (aPDT) plus KI.

Group	TBO Concentration (μg/mL)	KI Concentration (mM)	Light Dose (J/cm^2^)
Light	4	25, 50, 100, 200	24
Dark	4	25, 50, 100, 200	/

**Table 2:** Parameters for varying TBO concentration on TBO-mediated aPDT plus KI.

Group	TBO Concentration (μg/mL)	KI Concentration (mM)	Light Dose (J/cm^2^)
Light	0.25, 0.5, 1, 2, 4	200	24
Light	0.25, 0.5, 1, 2, 4	/	24
Dark	0.25, 0.5, 1, 2, 4	200	/
Dark	0.25, 0.5, 1, 2, 4	/	/

**Table 3:** Parameters for varying light dose on TBO-mediated aPDT plus KI.

Group	TBO Concentration (μg/mL)	KI Concentration (mM)	Light Dose (J/cm^2^)
1	4	200	24
2	4	/	24

## Data Availability

The data presented in this study are available on request from the corresponding author.
